# Does Long-Term High Fat Diet Always Lead to Smaller Hippocampi Volumes, Metabolite Concentrations, and Worse Learning and Memory? A Magnetic Resonance and Behavioral Study in Wistar Rats

**DOI:** 10.1371/journal.pone.0139987

**Published:** 2015-10-08

**Authors:** Zuzanna Setkowicz, Agata Gaździńska, Joanna J. Osoba, Karolina Karwowska, Piotr Majka, Jarosław Orzeł, Bartosz Kossowski, Piotr Bogorodzki, Krzysztof Janeczko, Mariusz Wyleżoł, Stefan P. Gazdzinski

**Affiliations:** 1 Department of Neuroanatomy, Jagiellonian University, Krakow, Poland; 2 Nencki Institute of Experimental Biology, Polish Academy of Sciences, Warsaw, Poland; 3 Mossakowski Medical Research Centre Polish Academy of Sciences, Warsaw, Poland; 4 Faculty of Electronics and Information Technology, Warsaw University of Technology, Warsaw, Poland; 5 Military Institute of Aviation Medicine, Warsaw, Poland; University of Palermo, ITALY

## Abstract

**Background:**

Obesity is a worldwide epidemic with more than 600 million affected individuals. Human studies have demonstrated some alterations in brains of otherwise healthy obese individuals and elevated risk of neurodegenerative disease of old age; these studies have also pointed to slightly diminished memory and executive functions among healthy obese individuals. Similar findings were obtained in animal models of obesity induced by high fat diet. On the other hand, low carbohydrate high fat diets are currently promoted for losing weight (e.g., Atkin’s style diets). However, the long-term effects of such diets are not known. Additionally, high fat diets leading to (mild) ketonemia were shown to improve brain function in elderly humans and in some animal models.

**Aim:**

To evaluate the hypothesis that long-term use of a high fat diet was associated with decreases in spatial memory, smaller hippocampi and hippocampi metabolite concentrations in Wistar rats.

**Methods:**

Twenty five male Wistar rats were put on high fat diet (HFD; 60% calories from fat, 30% from carbohydrates) on their 55^th^ day of life, while 25 control male rats (CONs) remained on chow. Adequate levels of essential nutrients were provided. Both groups underwent memory tests in 8-arm radial maze at 3^rd^, 6^th^, 9^th^, and 12^th^ month. ^1^H magnetic resonance spectroscopy was employed to measure concentrations of tNAA (marker of neuronal integrity) at one month and one year, whereas MRI was used to evaluate hippocampal volumes.

**Results:**

Obese rats (OBRs) consumed similar amount of calories as CONs, but less proteins. However, their protein intake was within recommended amounts. Throughout the experiment OBRs had statistically higher concentrations of blood ketone bodies than CONs, but still within normal values. At post-mortem assessment, OBRs had 38% larger fat deposits than CONs (p<0.05), as evaluated by volume of epididymis fat, an acknowledged marker of fat deposits in rats. Contrary to our expectations, OBRs had better scores of memory behavioral tasks than CONs throughout the experiment. At one year, their hippocampi were by 2.6% larger than in CONs (p = 0.05), whereas concentration of tNAA was 9.8% higher (p = 0.014).

**Conclusion:**

Long-term HFD in our study resulted in better memory, larger hippocampal volumes, as well as higher hippocampal metabolite concentrations, possibly due to increased levels of blood ketone bodies. The results should be interpreted with caution, as results from animal models do not necessarily directly translate in human condition.

## Introduction

Obesity is a disorder characterized by excessively high amount of body fat. Worldwide prevalence of obesity has reached epidemic proportions. In 2014, more than 1.9 billion adults, 18 years and older, were overweight. Of these over 600 million were obese. Once considered a problem only in high-income countries, obesity is now dramatically on the rise in low- and middle-income countries (WHO fact sheet No 311, updated January 2015). Human studies demonstrate some alterations in brains of otherwise healthy obese individuals (e.g., [1]). On general population level, otherwise healthy obese and overweight humans demonstrate worse performance on tests of learning, memory, and executive functions than their counterparts at healthy weight [2, 3]. Furthermore, increase in BMI during lifetime has been associated with decline in executive functions and memory [4, 5]. However, improvement in spatial abilities with increasing BMI was noted [5].

Neuroimaging studies also demonstrate brain alterations in otherwise healthy obese individuals. Our proton magnetic resonance spectroscopy (^1^H MRS) of healthy middle-aged and elderly individuals showed that higher BMI was related to lower absolute concentrations of tNAA throughout the white matter and in the frontal gray matter [6, 7]. tNAA consists of N-acetylaspartate and N-Acetylaspartylglutamate. In human research tNAA is an accepted marker of neuronal viability, observed only in mature neurons and their processes; decreased tNAA reflects neuronal loss, atrophied dendrites and axons, and/or derangements in neuronal energetics [8]. tNAA concentrations are lowered consistently throughout neurodegenerative and psychiatric diseases, such as Alzheimer’s disease, epilepsy, and alcohol dependence.

Obesity epidemic was triggered by is excessive caloric consumption [9]. On the other hand, epidemiological studies were not able to conclusively identify nutritional factors leading to poorer cognition [10]; even the role of dietary fat in cognitive decline is not clear (e.g., [11]). Nonetheless, animal studies using high fat diets (HFD) have generally demonstrated detrimental effects of these diets on memory and hippocampal morphology [12, 13], although glucose treatment [14], environmental enrichment [15], or physical activity [16] partially mitigated their effects. It is important to note that fat content in HFD by weight ranges from 10% [17] to 40% [18]. A study by Auer and colleagues [16] demonstrated that HFD led to lower concentrations of hippocampal metabolites including tNAA. A recent study in Cockayene syndrome mice demonstrated that high fat diet (60% energy from fat) may delay aging processes [18]. In humans, low carbohydrate high fat diets are currently promoted for losing weight (Atkin’s style diets). In Poland, followers of so called Kwasniewski diet (more than 70% energy intake from fat) demonstrated normal blood fasting glucose, insulin, HDL, triglycerides, blood pressure among, with elevated LDL [19] (long-term effects of this diet were not evaluated). Similarly, some studies demonstrated similar effects of Eskimo diet that contains large amounts of fat from animal origin [20, 21]. However, it should be noted that Eskimo population has broader access to other diets nowadays [22]. Additionally, high fat diets leading to mild ketonemia were shown to improve brain function in the elderly [23], whereas ketogenic diets lead to cognitive improvements in animal models and in patients suffering from drug-resistant epilepsy [24]. Unfortunately, the levels of blood ketone bodies were no evaluated in majority of studies (on animal) models claiming detrimental effects of high fat diet on the brain and its function.

Here, we utilized three complementary methods to evaluate long-term effects of HFD on the brain and its function in Wistar rats. A radial maze was used to measure spatial memory in rats, MRI was utilized to evaluate hippocampal volumes *in vivo*. Additionally, proton magnetic resonance spectroscopy (^1^H-MRS) at short echo time was applied to measure the concentrations and their changes due to the dietary treatment of the major brain metabolites: [8]: (1) tNAA (see above); (2) glutamate (Glu), an important molecule in cellular metabolism mostly found within neurons; it is also the main excitatory neurotransmitter and a potential marker of neuronal viability; striatal Glu levels were related to tNAA concentrations and neurocognition; its concentration can be reliably measured in field strengths of 3T and higher, (3) choline-containing compounds (tCho), which are believed to be primarily involved in cell membrane breakdown and synthesis; (4) creatine-containing metabolites (tCr) involved in cell bioenergetics, and (5) myo-inositol (Ino) that is considered a marker of glial cell numbers and an osmoregulator. Finally, we compared concentrations of taurine (Tau), an osmoregulator, and gamma-aminobutyric acid (GABA), major inhibitory neurotransmitter between groups. These measures were selected because they are often used in human studies of aging.

We evaluated the hypothesis that long-term use of high fat diet leads to decreases in spatial memory and smaller hippocampi and hippocampi metabolite concentrations in Wistar rats.

## Results and Discussion

### Breeding

**Fig 1:**
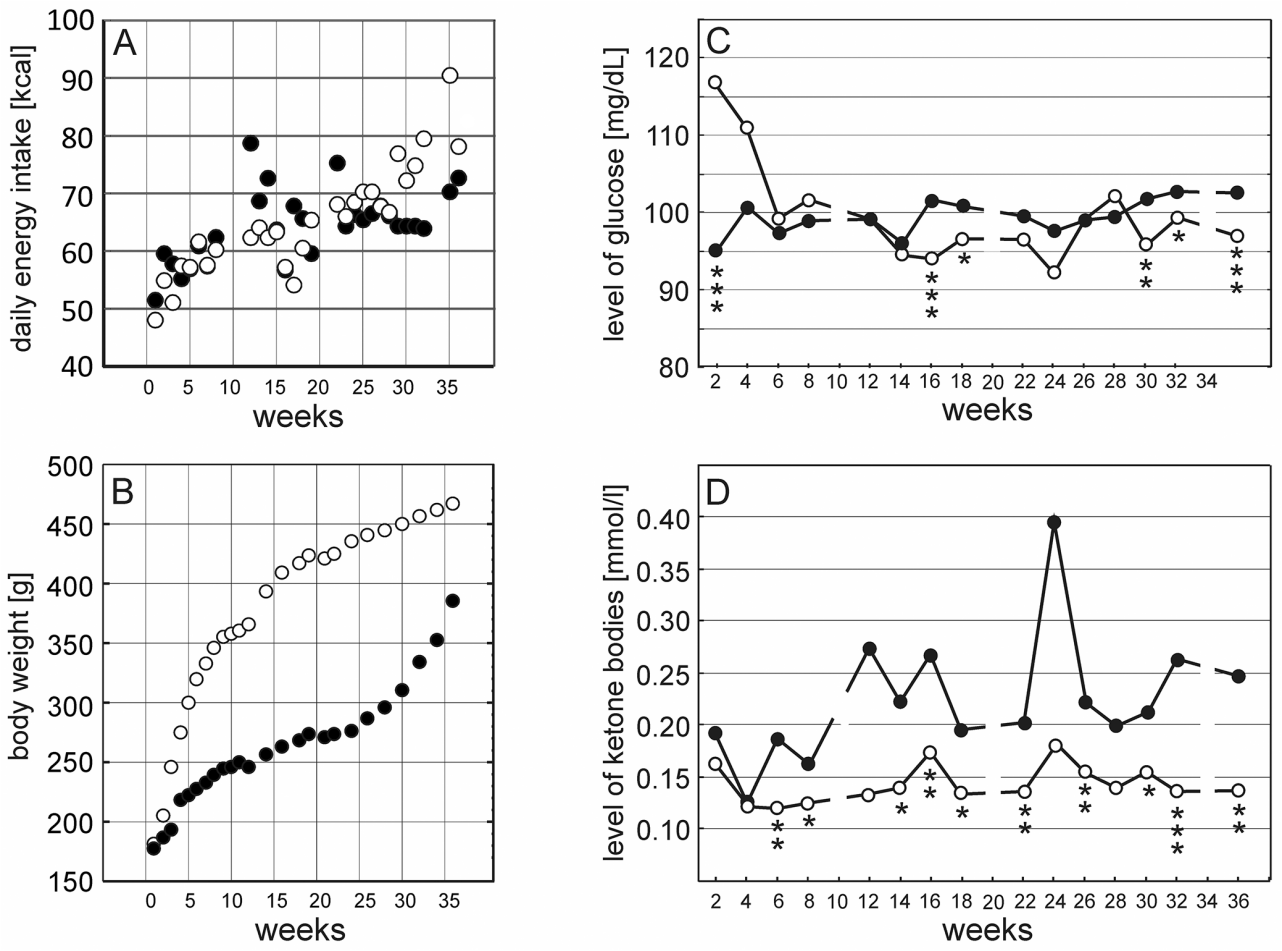
Daily energy intake (A), changes in body weight during the experiment (B), changes in glucose blood levels (C), and changes in blood levels of ketone bodies (D) in controls and HFD-fed rats. *P<0.05, ***P<0.01. The “spikes” in levels of ketone bodies were caused by short-lived increases in concentrations of ketone bodies in single animals.

Daily energy intake, body mass changes throughout the experiment, blood glucose levels and concentrations of blood ketone bodies are depicted in Fig 1.

Groups had similar caloric intake and OBRs had similar body mass as CONs, consistent with previous studies that did not observe differences in body mass between obese and control groups (e.g., [25–27]). However, at the time of being sacrificed, OBRs had 38% more epididymis fat (p<0.05, n = 32), an accepted marker of fatness in rats [28]. It demonstrates that OBRs had larger body fat depositions than the CONs, consistent with the planned effects of the diet. Blood level of sugar in OBRs was significantly elevated after 8th month of the experiment, but still within normal values. Blood concentration of ketone bodies were elevated throughout the experiment in OBRs compared to CONs (0.22±0.04mM vs. 0.14±0.03mM), reaching the range of mild ketonemia [29]. At the time of being sacrificed, OBRs had 5.8% heavier brains than CONs (1.80g vs. 1.63g, p<0.01). The raw data is provided in S1 Table.

### Memory

**Fig 2:**
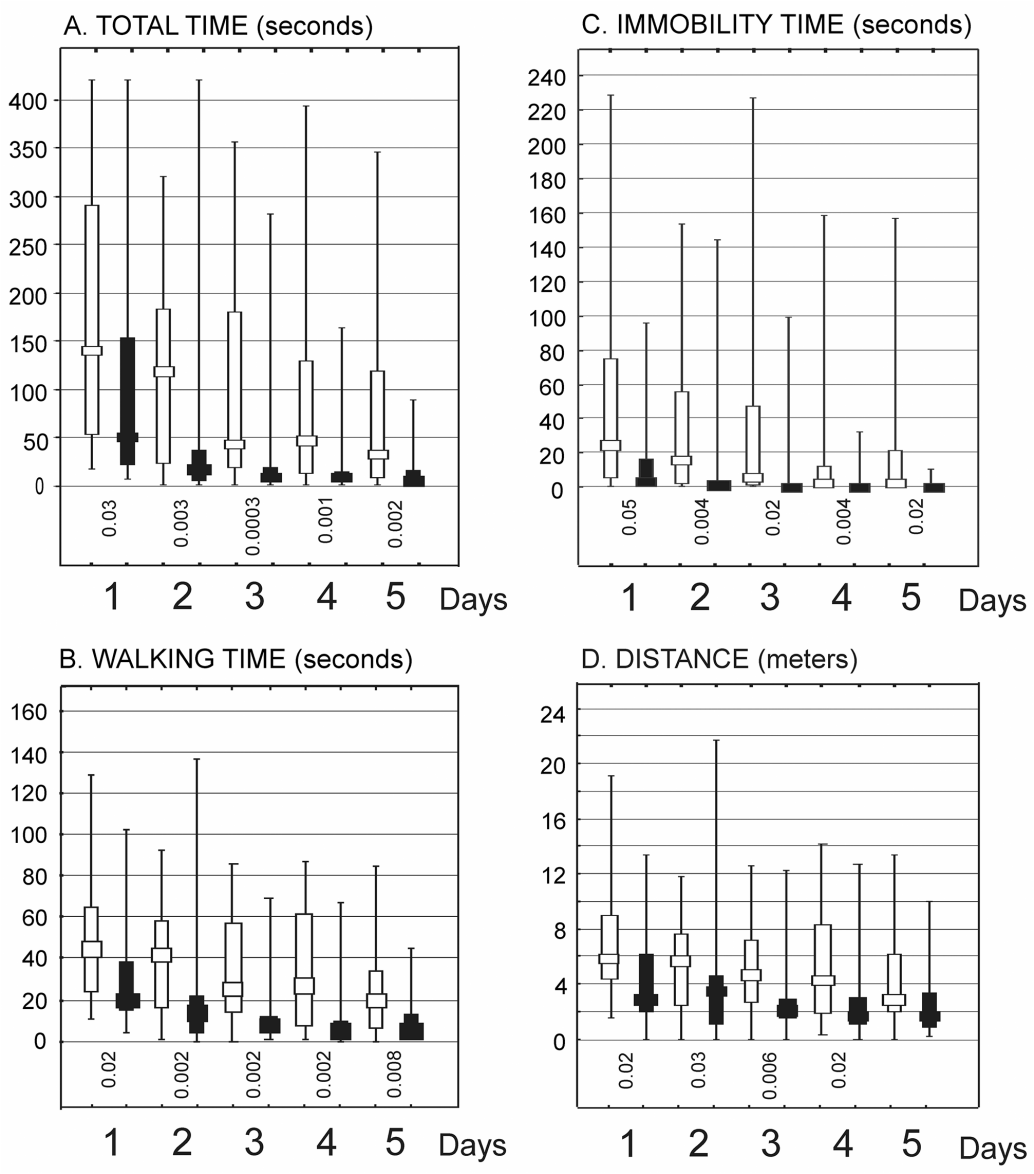
Exemplary parameters of the animal behavior during five-day maze tests performed/repeated at the 9^th^ month of age. (A) the total time (seconds) spent in the maze before the reward was reached by the animal on day 1^st^, 2^nd^, 3^rd^, 4^th^, and 5^th^, (B) the time of walking (seconds) to reach the reward, (C) the time of immobility (seconds), (D) the total walking distance (meters). Abbreviations: White and black graphs characterize control and high-fat high carbohydrate diet-treated animals, respectively. The graphs show median values (squares), 25–75% variability ranges (vertically arranged rectangles), and maximal and minimal values (whiskers). Decimal indexes located below the graphs indicate statistical significance of intergroup differences at a given maze test (Mann–Whitney U-test).

Data used in the analyses is provided in S2 Table. Generally, OBRs had better memory than CONs. At every control stage of the experiment they were able to find the reward faster. The differences in the total time spent in the maze before the reward was reached by the animal were statistically significance at the 6th and 9th and 12^th^ month of life (see Fig 2). A similar pattern was found for time of walking (movement). Moreover, the vast majority of tests performed at the age of three, six and nine months detected significantly shorter average periods of immobility in OBRs. Significantly shorter walking distance characterized OBRs at the age of 9 months (Fig 2).

### Hippocampal volumes

**Fig 3:**
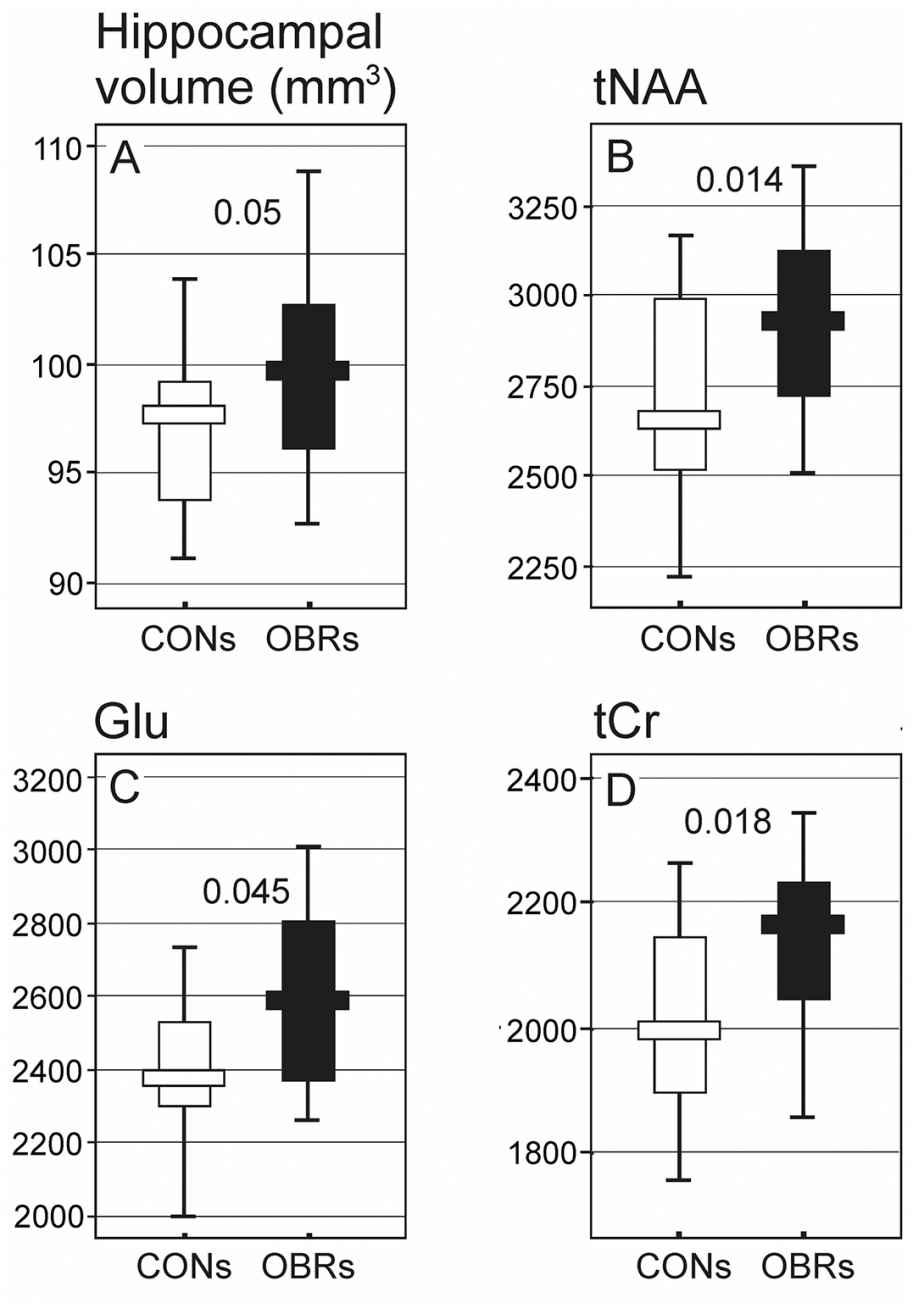
Hippocampal volumes (A) and concentration of metabolities: (B) N-acetylaspartate+N-acetylaspartylglutamate (tNAA), (C) glutamate (Glu) and (D) creatine+phosphocreatine (tCr). White and black graphs represent control (CONs) and high-fat high carbohydrate diet-treated (OBRs) animals, respectively. The graphs show median values with 25–75% variability range and maximal and minimal values. Decimal indexes indicate statistical significance of intergroup differences (Mann–Whitney U-test).

At one year, there was no significant difference in the absolute volume of the brain or the total cortex between the two groups (p>0.70). However, contrary to our hypotheses, the average hippocampal volume was 2.6% larger in OBRs than in CONs (p = 0.05, Fig 3A). For hippocampal volumes normalized to individual brain volumes, there was a trend for on average 2.1% larger hippocampi volume in OBRs than in CONs (p>0.05). No changes were found in the remaining 50 regions of the brain. Data used in these analyses is provided in S3 Table.

### Metabolite concentrations

Contrary to our hypothesis, the concentration of tNAA and Glu were respectively (9.8%, p = 0.014 and 8.6%, p = 0.045) higher in OBRs than in CONs (Fig 3B and 3C, respectively). Additionally, tCr was 8.2% higher in OBRs than in CONs (p = 0.045, Fig 3D). There were no differences in tCho, Ino, Tau and GABA concentrations (p>0.05).

These differences were not premorbid, as OBRs had significantly larger lifetime increases in tNAA and tCr than CON between 1^st^ (before switching to HFD) and 12^th^ month (p<0.01). Data used in all the above analyses is provided in S3 Table.

## Summary of Findings and General Discussion

In this study, the high-fat diet lead to mild ketonemia, small blood glucose elevations, as well as it better behavioral scores in OBRs throughout lifetime, larger hippocampi and higher concentrations of tNAA and Glu, the marker of neuronal viability and the main excitatory neurotransmitter, respectively.

Larger fat deposits at similar body weight in OBRs than in CONs point to smaller lean body mass, which is consistent with studies on the effects of ketogenic diets [30] or diets inducing mild ketonemia [29]. Better memory in OBRs is consistent with studies of the effects of ketogenic diets on memory (see [24]). Ketogenic diet increases glucose and ketone bodies uptake in the brain of aged rats [31, 32]. Moreover, elevated ketone levels were reported to be positively correlated with memory performance [23, 24]. It has to be stressed that future studies designed to evaluate the long-term effects of mild ketonemia on the brain and its function are necessary.

Our results show similarity to a study by Patten and colleagues [33], who observed that polyunsaturated fatty acids provided by the diet for 11 months improved the learning ability by enhancing synaptic plasticity in the hippocampus. Similarly to our study, the animals fed the high-calorie diet spent much less time and ran shorter distances in the maze than the control group. Consistent with it, Gunstad and colleagues demonstrated that increase in body mass index (BMI) in humans was associated with improvement in spatial abilities [5] that are likely hippocampal-dependent. Additionally, a high-fat diet (>70% caloric intake from fats) in a Polish nutritional sect did not lead to elevation in triglycerides and LDL to HDL ratio, insulin resistance or systemic inflammation [19]. Similar findings were reported in Eskimo population [20, 21]. Consistent with this, we observed lowered expression of gliosis-associated markers (markers of inflammation) in hippocampi in OBRs [34].

Furthermore, Pancani et al. [35] did not observe any deterioration of learning in a water maze in F344 rats fed high fat diet for 4.5 months. Recently, Beilharz and colleagues [36] showed that a short-term diet (from 5 till 21 days) rich in fats and sugars did not impair the ability to recognize new objects in rats. Thus, the better memory in OBRs might result from an increased level of cholesterol following this type diet application. However, a large body of studies providing smaller percent of energy from fat reported detrimental effects of high-fat diet on memory in animal models [13, 17, 37–41], possibly due to larger proportion of saturated and trans-fats, or increased caloric intake. In particular, Molteni with colleagues [38] reported that diet rich in saturated fats and refined sugars impaired spatial learning and lead to certain neurochemical abnormalities in the hippocampal formation, which is one of the brain areas most involved in learning and memory{Winocur, 1999 #796}. However, glucose administration improved spatial memory at least short-term [14].

Thus, prospective studies evaluating the effects of nutrition and physical activity on cognitive functions and markers of neurodegeneration are needed. They should also measure the concentrations of blood ketone bodies and clarify the effects for factors not accounted for in this study, such as potential insulin resistance, the potential role of gut microbiota [42] and genetic differences between individual specimens [43].

## Conclusion

This study provides consistent evidence (independent methodologies) that a high fat diet may have positive effects on the hippocampus and its function, as measured with behavior, MRI volumetry, and magnetic resonance spectroscopy. Caution should be exercised when attempting to generalize our results to humans. Non-dietary factors, such as stress, insufficiency of physical activity, lack of involvement/withdrawal from life activities, depressive symptoms common in obese humans may underpin the link between human obesity and neurodegenerative disease in old age may mediate the link between excessive caloric intake, high fat diet, obesity and poorer cognitive performance in humans. Prospective studies designed to evaluate the long-term effects of mild ketonemia on the brain and its function are necessary.

## Materials and Methods

### Animals

Male Wistar rats aged 45–50 days were randomly divided into two groups, i.e. rats fed HFD (OBRs) and controls (CONs) fed standard control diet (Labofeed-Morawski, Poland). By weight, the HFD consisted of about 36.5% lard (47% saturated fats, 49% mono- and poly unsaturated fats), 36.9% sucrose, 14.0% proteins, 4.0% vitamins, 1% cellulose, 3% starch/dextrin, essential minerals and trace elements; in terms of energy: 61% came from fats, 28% from sugars, and 11% from proteins. The control diet (4.7% lard; 37.0% starch/dextrin, 25.3% proteins, 4.0% vitamins, 3.9% fiber, 3.2% ash and essential minerals and trace elements) was used throughout the experiment; it translates to 14% energy from fats, 51% from carbohydrates, and 35% from proteins. Smaller protein content in HFD than in the control diet is consistent with human [44, 45] and some animal studies (e.g., [46]).

The animals were housed under conditions of controlled temperature (21 ± 2°C) and illumination (12-h light/dark cycle) in individual cages with unlimited voluntary access to food and water. Every three days food portions that were delivered to the cages and those left uneaten by the animals were weighed to calculate food intake. The animals were weighed every two weeks and the blood levels of ketone bodies and glucose were measured every week in the beginning of the dark phase (with Optium Xido glucometer, Abbott Diabetes Care Ltd., Oxon, UK), excluding weeks/days when behavioral tests were performed) over the period of 12 months [47]. After all experiments the animals were sacrificed with by a lethal dose of pentobarbital and their brain were prepared for post-mortem analyses. The results will be published elsewhere.

All procedures involving the use of animals were approved by the Bioethical Commission of the Jagiellonian University in Krakow, Poland, in accordance with international standards.

### Behavioral tests

Animals from the two examined groups were subjected to memory test in a radial eight arm maze at 3^rd^, 6^th^, 9^th^, and 12^th^ month of life; each procedure lasted for five days. One day before the training, the food rations were reduced to the half of daily demand according to established procedures [48]. The animals were habituated to the experimental conditions by placing them into the maze for 10 minutes per day during subsequent five days without any reward.

Thereafter, the spatial learning and memory was performed once on each of five consecutive days. A small piece of unsalted and unsweetened crunchy corn was a reward for both control and experimental groups. Following behavioral tests performed before the experiment, this kind of reward was selected from among the other types of food such as chocolate, cakes, candies, fruits, meat and living or dead insects, as there were no effects of diet on performance within a few weeks after introducing HFD. The reward was always placed in the same arm of the maze and the tested animal was placed in the maze center. The experimenter was out of sight of the animal, hidden behind white walls surrounding the maze and showing no visual markers. Behavior was recorded using the system Videomex-One (Columbus Instruments, Columbus, OH, USA) and the following parameters were automatically calculated: (1) entire time spent in the maze, (2) time of walking (movement), (3) time of immobility, and (4) distance covered by the animal. All trials were finished within 10 minutes.

### Magnetic Resonance image acquisition and processing

At one month and at 12th month, brains of the examined animals were scanned with Bruker BioSpec 70/30 Avance III system working at 7T, with a transmit cylindrical radiofrequency coil (15 cm inner diameter) and a receive-only coil array (2x2 elements) positioned over the animal’s head. The animals were positioned prone with the head placed in the stereotactic apparatus and were anesthetized with 1.5% isoflurane in a mixture of oxygen and air. Respiration, heart rate, and oxygen saturation were monitored throughout the experiment. Rectal temperature was kept at 37°C by placing the animal on top of temperature controlled warm water blanket. Tripilot scans were used for accurate positioning of the animals inside the magnet.

Structural MR images covering the brain without olfactory bulb were acquired with T2-weighted TurboRARE (TR/TE = 4700/30ms, RARE factor = 4, resolution = 125x 125x500μm, no gap, NEX = 7, TA = 27 min) for brain parcellation (see below).

**Fig 4:**
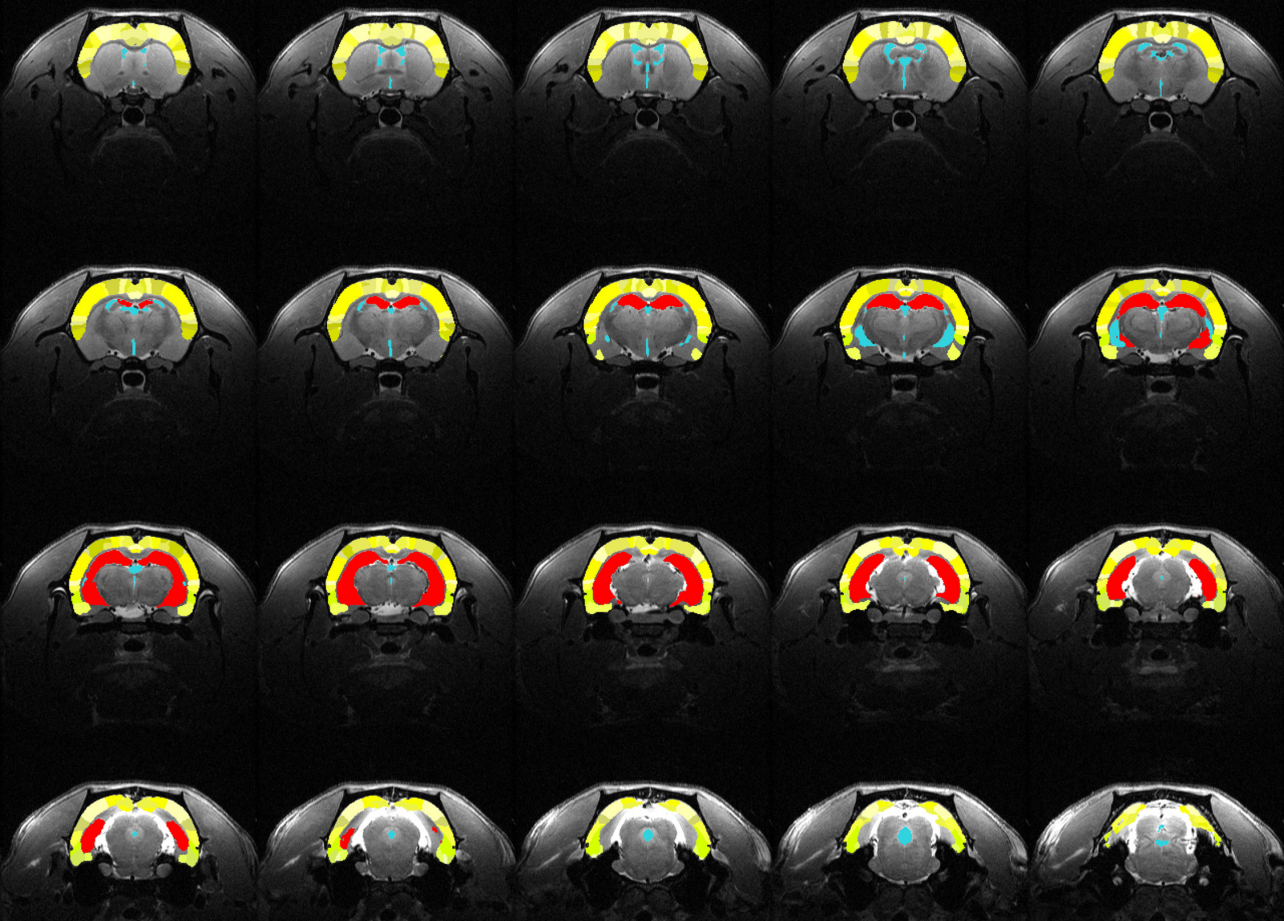
Exemplary parcellation of hippocampus: red = hippocampus, yellow = cortex, blue = cerebro-spinal fluid.

MR images were resampled to isotropic resolution of 125μm/vox and then processed with N4 algorithm [49] to correct for intensity inhomogenity. Afterwards, a single image was chosen randomly and the brain outline was drawn semi automatically using the ITK-Snap software [50] and transferred to the other images using the Symmetric normalization (SyN) nonlinear image registration algorithm (ANTS software package [51]. Skull stripped brain images were then parceled by warping to the template proposed by Valdes-Hernandez et al. [52] that included 50 brain, mostly cortical regions. This template was extended by manual parcellation of the hippocampus (PM). All parcelled images were visually reviewed to assure quality. Exemplary parcellation of hippocampi is depicted in Fig 4. Absolute volumes were reported. Additionally, to account for intersubject variability, hippocampal volumes were scaled to the whole brain and compared between groups.

### ^1^H Magnetic Resonance Spectroscopy acquisition and processing

**Fig 5:**
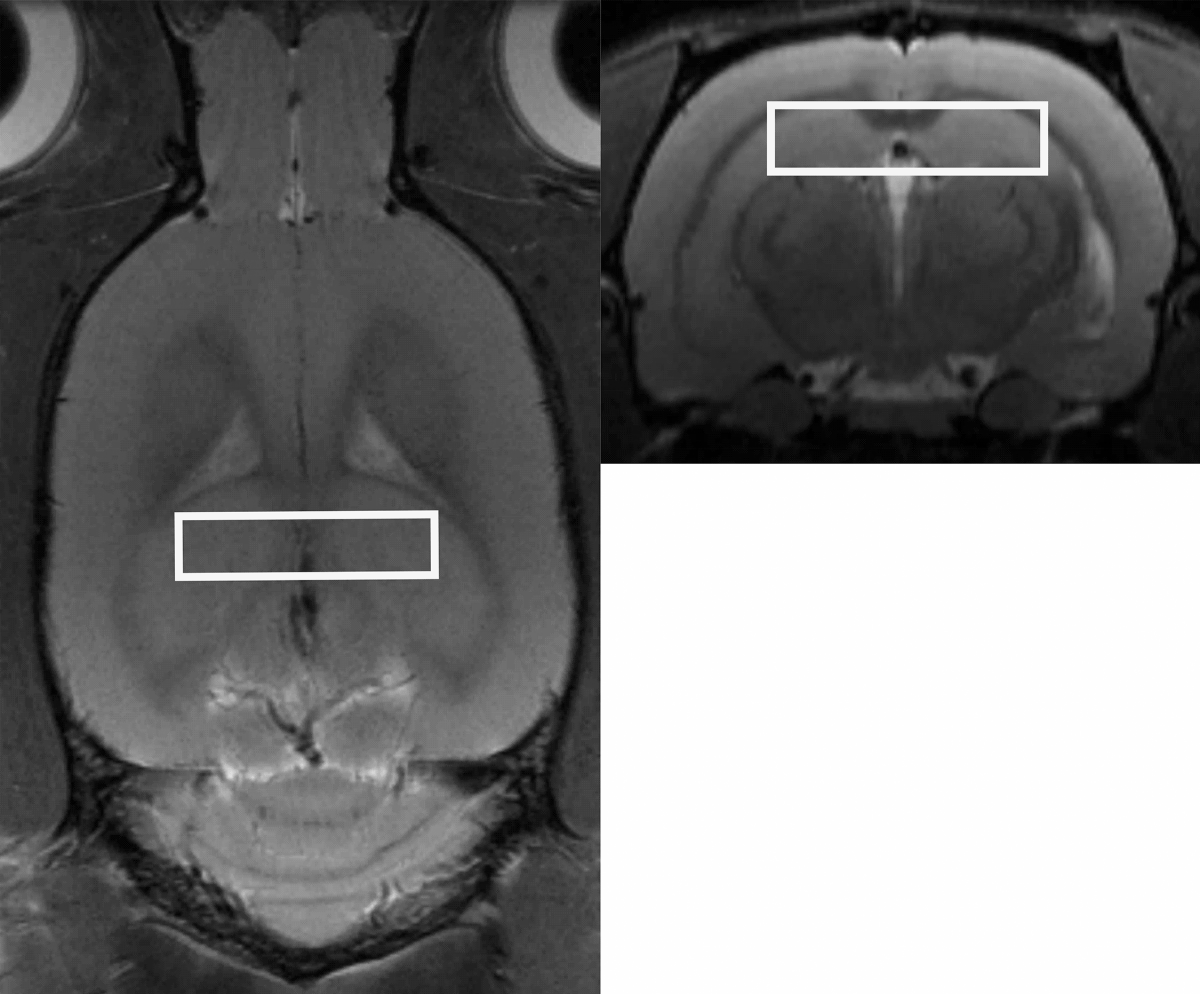
Volume of interest placement.

**Fig 6:**
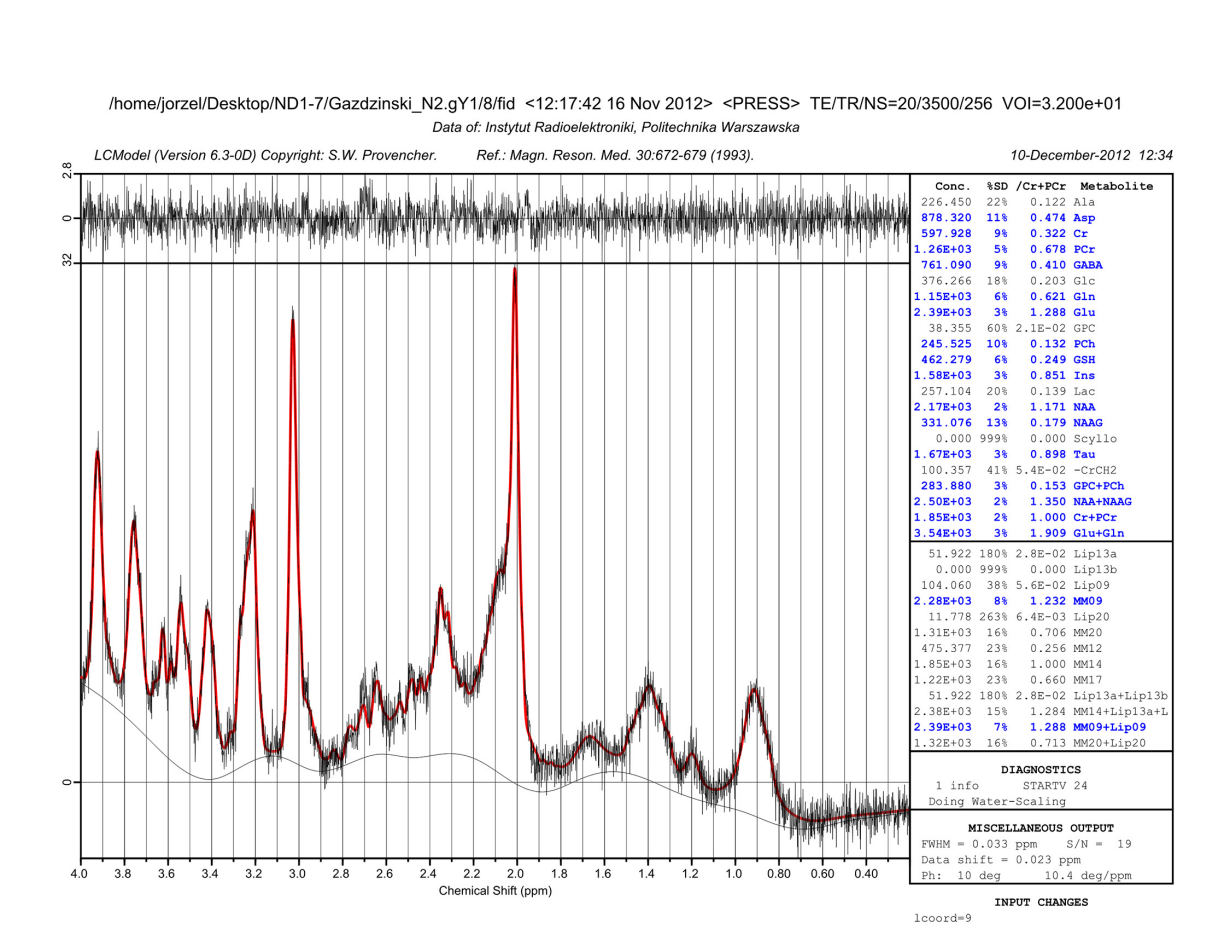
Exemplary proton spectrum with fitted baseline.

To obtain the spectra of brain metabolites, localized proton spectroscopy at short echo was performed using PRESS sequence (TR/TE = 3500/20 ms, 256 averages, 8,192 points, TA = 15min) with VAPOR water suppression, the outer volume suppression, and frequency drift correction (flip angle 7 deg.). No eddy current correction was performed at this step. Each measurement was carried out in a single volume of interest (8 x 2 x 2 mm^3^) encompassing hippocampus (Fig 5). The FASTMAP shimming procedure was performed. The unsuppressed water line width was typically maintained at around 10–15 Hz. Metabolite concentrations were determined using a linear combination analysis method LCModel (Stephen Provencher Inc, Oakville, Ontario, Canada; [53] (Fig 6). The unsuppressed water signal measured from the same volume of interest was used as internal reference for absolute metabolite quantification. Metabolite concentrations are reported in institutional units (i.u.).

All spectra were visually reviewed for quality. Only metabolites that demonstrated good reliability on test-retest [54] were used in analyses: tNAA (N-Acetylaspartate + N-Acetylaspartylglutamate), tCr (Creatine + Phosphocreatine), tCho (Glycerophosphocholine +Phosphocholine), Ins (myo-Inositol), GABA (γ-Aminobutyric Acid), and Tau (Taurine).

### Statistical analyses

Due to non-Gaussian distribution of parameters, median values and non-parametric Mann-Whitney U-test are reported. Per cent differences between groups were calculated based on medians (imaging data, physiology, but not behavior). Hippocampal volumes had Gaussian distribution and a parametric t-test was used. For not hypothesized contrasts Bonferroni corrections were applied. A significance level of p<0.05 (after correction for multiple comparisons) was considered statistically significant. In follow-up analyses, relationships between measures were evaluated using appropriate statistics. All statistical tests were conducted with SPSS-21 for Windows (SPSS; Chicago, IL).

## Supporting Information

S1 Table Physiology (data on breeding). (XLSX)

S2 Table Behavior. (XLSX)

S3 Table MRI and MRS data for spectroscopy and volumetric analyses (XLSX)
